# Flocculation of *Chlamydomonas reinhardtii* with Different Phenotypic Traits by Metal Cations and High pH

**DOI:** 10.3389/fpls.2017.01997

**Published:** 2017-11-20

**Authors:** Jianhua Fan, Lvhong Zheng, Yunpeng Bai, Shai Saroussi, Arthur R. Grossman

**Affiliations:** ^1^State Key Laboratory of Bioreactor Engineering, East China University of Science and Technology, Shanghai, China; ^2^Department of Applied Biology, East China University of Science and Technology, Shanghai, China; ^3^Department of Plant Biology, Carnegie Institution for Science, Stanford, CA, United States

**Keywords:** flocculation, cell wall deficient, Chlamydomonas, multivalent metal ions, microalgae, pH, algal biotechnology

## Abstract

Concentrating algal cells by flocculation as a prelude to centrifugation could significantly reduce the energy and cost of harvesting the algae. However, how variation in phenotypic traits such as cell surface features, cell size and motility alter the efficiency of metal cation and pH-induced flocculation is not well understood. Our results demonstrate that both wild-type and cell wall-deficient strains of the green unicellular alga *Chlamydomonas reinhardtii* efficiently flocculate (>90%) at an elevated pH of the medium (pH 11) upon the addition of divalent cations such as calcium and magnesium (>5 mM). The trivalent ferric cation (at 10 mM) proved to be essential for promoting flocculation under weak alkaline conditions (pH ∼8.5), with a maximum efficiency that exceeded 95 and 85% for wild-type CC1690 and the cell wall-deficient *sta6* mutant, respectively. Near complete flocculation could be achieved using a combination of 5 mM calcium and a pH >11, while the medium recovered following cell removal could be re-cycled without affecting algal growth rates. Moreover, the absence of starch in the cell had little overall impact on flocculation efficiency. These findings contribute to our understanding of flocculation in different Chlamydomonas strains and have implications with respect to inexpensive methods for harvesting algae with different phenotypic traits. Additional research on the conditions (e.g., pH and metal ions) used for efficient flocculation of diverse algal groups with diverse characteristics, at both small and large scale, will help establish inexpensive procedures for harvesting cell biomass.

## Introduction

Over the past decade there has been an increasing interest in microalgae as a promising feedstock for sustainable, large-scale production of commodities such as food, feed, chemicals, materials and fuels (Abomohra et al., 2016; Waghmare et al., 2016). For all of the products that could potentially be developed, overcoming technical issues and reducing production costs will be the primary determinants of feasibility for achieving sustainable algal biomass production (Laurens et al., 2017). Among key technical issues, a major challenge for commercial-scale applications is the ability to inexpensively harvest large quantities of microalgal biomass from dilute cultures. Conventional methods like centrifugation are fast and effective, but also costly and energy intensive, making it only suitable for the production of high-value biomass or metabolites (Demirbas, 2017). The various other techniques that have been used to harvest microalgae include membrane filtration (Lorente et al., 2017), foam fractionation (Ndikubwimana et al., 2016a), chemical/biological flocculation (Chatsungnoen and Chisti, 2016; Ndikubwimana et al., 2016b), electrolytic coagulation (Fayad et al., 2017), ultrasonic aggregation (Wang et al., 2015), magnetic separation (Safarik et al., 2017) and gravity sedimentation (Griffiths et al., 2012). Each of these methods has been reported, with some promising results under specific production conditions.

To generate algal biomass in an economically feasible, environmentally friendly way requires selection of appropriate harvesting technologies. Several factors must be considered when selecting the harvesting strategy, including strain phenotype, ionic strength and pH conditions of the culture medium, recycling of spent medium, and the final quality of harvested biomass. Chemical flocculation with polyvalent metal ions or polymeric flocculants is widely used in water treatment and has been shown to efficiently separate microalgal cells from its growth medium (Chatsungnoen and Chisti, 2016). For some species, flocculation can also be stimulated by changing the pH of the medium. However, increasing the pH can lead to precipitation of magnesium, calcium, phosphate and carbonate salts along with the algal cells (Vandamme et al., 2012; Wu et al., 2012). In contrast, decreasing the pH neutralizes negative charges on the cell surface, which decreases dispersal forces among the cells thereby promoting flocculation; the flocculated material can then be collected in a much smaller total culture volume (Liu et al., 2013) and be further concentrated by centrifugation. It was recently shown that the power consumption during the harvesting of microalgae by coagulation flocculation was much lower relative to the power consumption of conventional centrifugation [only 2.1 kWh/kg for *Chlorella vulgaris* and 0.2 kWh/kg for *Phaeodactylum tricornutum* (Vandamme et al., 2011) compared to ∼16 kWh/kg when assuming a microalga biomass concentration of 0.5 kg/m^3^ in open ponds (Danquah et al., 2009)]. In sum, algal cell flocculation prior to harvesting by centrifugation could significantly reduce the energy ‘cost’ of the collection procedure (Xu et al., 2011).

Although it appears from previous studies that flocculation is an efficient technique to pre-harvest microalgal cells, there is still uncertainty about how general the procedure is for different species and for diverse strains of a single species that are phenotypically distinct, which would include differences in cell size, surface charge properties and motility. It would also be valuable to explore how cation and pH triggered flocculation is impacted when the production strains are lacking or deficient in a cell wall; such strains may be more amenable to molecular and genetic engineering as well as cell disruption.

To further develop simple, inexpensive methods to facilitate algal cell harvesting by flocculation, we evaluated flocculation of the green unicellular algae *Chlamydomonas reinhardtii* (hereafter Chlamydomonas) using a combination of metal cations in addition to medium of different pHs. Different strains with different physical and biological properties (cell wall, size, motility, etc.) were examined in these studies to determine if specific physical characteristics impact the extent or efficiency of flocculation. We therefore analyzed several Chlamydomonas strains with different properties including two wild-type strains, CC124 and CC1690 (also known as 21gr^+^), that are widely used in the laboratory, and the cell wall deficient (*cw*^-^) strain *cw15 sta6* (BAFJ5, lack flagella), which was derived from strain CC330 by random insertional mutagenesis with the *ARG7*-containing plasmid (Zabawinski et al., 2001); *cw15 sta6* is unable to synthesize starch as a consequence of the *STA6* gene deletion and lacks motility as it does not have a flagellum (Wang et al., 2009). Since the *sta6* mutant displays normal growth rates (relative to wild-type cells) in acetate-supplemented medium but does not synthesize starch, many studies over the last two decades have explored carbon partitioning in this strain and its potential to synthesize high levels of lipids for the production of sustainable, renewable liquid fuels (Siaut et al., 2011; Blaby et al., 2013; Goodenough et al., 2014). In addition, we examined CC400, which is a *cw*^-^ strain that synthesizes starch, and a *sta6* strain (CC4567) genetically rescued for the starchless phenotype by transformation with a wild-type copy of the *STA6* gene [*sta6*::*STA6*, hereafter designated C6 (Li et al., 2010)]; these latter strains help distinguish the impact of starch accumulation and cell wall synthesis on flocculation. Overall, our findings challenge the idea that immobile strains that lack flagella and/or cell walls more readily flocculate, while at the same time furthering our understanding of flocculation in distinct Chlamydomonas strains. Similar experiments can now be performed with a range of microalgae that show promise for industrial applications.

## Materials and Methods

### Microalgae Cultivation

*Chlamydomonas reinhardtii* cells were cultured in sterile Tris-Acetate-Phosphate (TAP) (Chlamydomonas Resource Center^1^) and high salt (HS) medium (Sueoka, 1960) adjusted to pH 7.2. For all experiments the cells were grown in triplicate in Erlenmeyer Flasks at 25°C with shaking. The *C. reinhardtii* strains (Supplementary Table S1) used in these experiments were standard parental strains CC124 (137c *mt^-^ nit1 nit2*) and CC1690 (*mt*^+^ *NIT1 NIT2*), the cell wall deficient strain CC400 (*cw15*), the cell wall-deficient mutant with a lesion at the *STA6* locus CC4348 (*cw15 arg7-7 nit1 nit2 sta6-1::ARG7*) that is generally denoted *sta6*, and the *sta6* rescued strain CC4567, generally denoted *sta6*-C6, which was generated by transformating *sta6* with the plasmid pSL-*STA6*; this plasmid carries a genomic copy of the wild-type *STA6* gene (*cw15 arg7-7 nit1 nit2 sta6-1::ARG7 STA6*). These strains were obtained from the Chlamydomonas Resource Center. Before use in flocculation experiments, 5 mL of cells grown photoautotrophically in HS medium were inoculated into 100 mL of TAP medium in a 250-mL Erlenmeyer flask that was exposed to continuous light (100 μmol photons m^-2^s^-1^) with agitation (150 rpm) at 25°C for 8 days.

### Flocculation Experiments

Flocculation experiments were performed at an algal density of ∼0.7 g dry weight per liter. The effects of pH were examined for algal suspensions by either increasing or decreasing the pH with the addition of sodium hydroxide (NaOH) and hydrochloric acid (HCl) from 2 M stock solutions. Experiments were performed in 100-mL beakers that were magnetically stirred and mixed vigorously for 2 min during and immediately after pH adjustments. For metal cation treatments, 5 M stock solutions of ferric chloride (FeCl_3_), calcium chloride (CaCl_2_), and magnesium chloride (MgCl_2_) were prepared and each was added to the medium to generate final concentrations of 0, 1, 2.5, 5, 10, and 30 mM.

To evaluate flocculation, 20 mL aliquots of the cultures were collected from the beakers, transferred to disposable test tubes and incubated without agitation for 15 min. Following this potential ‘flocculation period,’ an aliquot of culture was withdrawn (from the middle of the ‘clarified zone’) and used to determine both OD_750_ and the absolute number of cells per mL. The flocculation efficiency was calculated according to the equation: flocculation efficiency (%) = (1-A/B) × 100 (where *A* represents cell number in the clarified zone and *B* the cell number of the reference, untreated control).

### Measuring Methods

Cell numbers were determined by counting intact cells using Countess II FL (Life Technology, United States). Microscopic snapshots were taken on a Leica optical microscope (LEITZ DMRB, Germany) which also enabled determination of cell diameter. A Malvern Zetasizer 2000HSA (Malvern, United Kingdom) was used to measure the zeta potential of the algal cultures in deionized water (cells were collected from the growth medium by centrifugation and resuspended in deionized water) (Liu et al., 2013), which relates to the surface charge of individual cells. For dry cell weight, the algal cells were collected by centrifugation at 12,000 *g* for 5 min, washed twice with distilled water, dried at 105°C for 24 h, and weighed to obtain total dry biomass. The dried cell powder was extracted with a solvent mixture of chloroform and methanol (2:1, v/v) to calculate the lipid content (Fan et al., 2014). The starch content was analyzed using a commercial enzymatic Starch Assay Kit (SA-20, Sigma–Aldrich). The concentrations of the metal cations in the spent medium were measured using inductively coupled-atomic emission spectrometry (ICP-AES). The operating conditions for ICP-AES instrument were as previously described (Matsuura et al., 2001). The measuring wavelength and atomic/ionic lines were as follows, Fe (259.9 nm, II), Ca (317.9 nm, II) and Mg (279.0 nm, II).

### Reuse of the Liquid Phase as Growth Medium

Following 30 min of coagulation-flocculation, spent supernatants were tested for their ability to support algal growth (media recyclability). The pH value of the spent medium was adjusted to approximately neutral by adding HCl, while macronutrients and micronutrients were added according to the TAP medium recipe, except that we did not include acetate and added CaCl_2_ to 50% of the level present in TAP medium. The adjusted supernatants were tested for their ability to sustain photoautotrophic growth of Chlamydomonas (25°C, 100 μmol photons m^-2^s^-1^, bubbled with 1% CO_2_).

### Statistical Analysis

All experiments were performed in triplicate (biological) and the data, presented as a mean ± standard deviation (*SD*), were further analyzed by Student’s *t*-test (*n* = 3). Asterisks indicate a significant difference from the control.

## Results and Discussion

### The Role of pH in Self-Flocculation for Different Chlamydomonas Strains

We first examined three different Chlamydomonas strains to determine whether different phenotypic characteristics [difference in cell wall, flagella and size (Zabawinski et al., 2001; Siaut et al., 2011); **Table 1**] impact the tendency of the cells to flocculate in response to various pHs and metal cations. The strains used were the ‘wild-type’ strains CC124 and CC1690, which contain a cell wall and flagella, but significantly differ in their genetic background (Gallaher et al., 2015), the starchless mutant, *sta6*, which has neither a cell wall nor a flagella (Zabawinski et al., 2001; Wang et al., 2009; Siaut et al., 2011) and its complemented strain, C6, as well as CC400 (**Table 1**). Eight days following inoculation, the cell densities of all of the cultures increased from 5 × 10^5^ to between 4 × 10^7^ and 9 × 10^7^ cell mL^-1^ (**Supplementary Figure S1**). All strains were spherical, although the average diameter of the *sta6* mutant was much smaller than those of the other strains; 6.29 ± 0.37 μm (CC124), 6.88 ± 0.43 μm (CC1690), and 2.29 ± 0.20 μm (*sta6*) (**Table 1** and **Supplementary Figure S2**). The maximal dry cell weight ranged from 0.63 g/L (for *sta6*, which attained a density of 8.81 × 10^7^) to 0.78 g/L (for CC1690, which attained a density of 4.53 × 10^7^); similar biomass yields of 0.5–1 g/L are considered typical for photoautotrophic cultivation of oleaginous algae in open raceway ponds (Rawat et al., 2013).

**Table 1:** Cell properties and flocculation characteristics using calcium ions at pH ∼11.5.

Strains	Cell wall and flagellar motility	Cell density (g/L)	Size (μm)	Starch content (%)	Lipid contents (%)	Optimized dosage (mM, CaCl_2_)	Flocculation efficiency (%)	Zeta potential (mV)	
								Before flocculation	After flocculation
CC1690	+	0.78 ± 0.07	6.89 ± 0.25	32.5 ± 2.3	11.2 ± 1.9	5	94.8 ± 2.3	–22.15 ± 1.69	–5.67 ± 0.38
CC124	+	0.74 ± 0.09	5.96 ± 0.37	39.2 ± 3.4	14.7 ± 2.3	5	92.9 ± 2.6	–20.48 ± 2.31	–6.43 ± 0.57
*sta6*	–	0.63 ± 0.09	2.33 ± 0.18	ND	45.3 ± 2.6	10	93.7 ± 1.9	–26.63 ± 2.04	–4.92 ± 0.27
CC400	–	0.68 ± 0.06	4.27 ± 0.24	29.4 ± 2.9	13.4 ± 2.7	10	94.4 ± 2.2	–22.49 ± 2.18	–3.73 ± 0.44
*sta6-*C6	–	0.66 ± 0.07	3.57 ± 0.21	33.5 ± 1.9	16.2 ± 3.3	10	94.1 ± 1.7	–25.43 ± 1.95	–4.67 ± 0.51

*+: intact cell wall and flagella-dependent motility; -: cell wall deficient and lacking flagella-dependent motility; ND, not detectable.*

**FIGURE 1:**
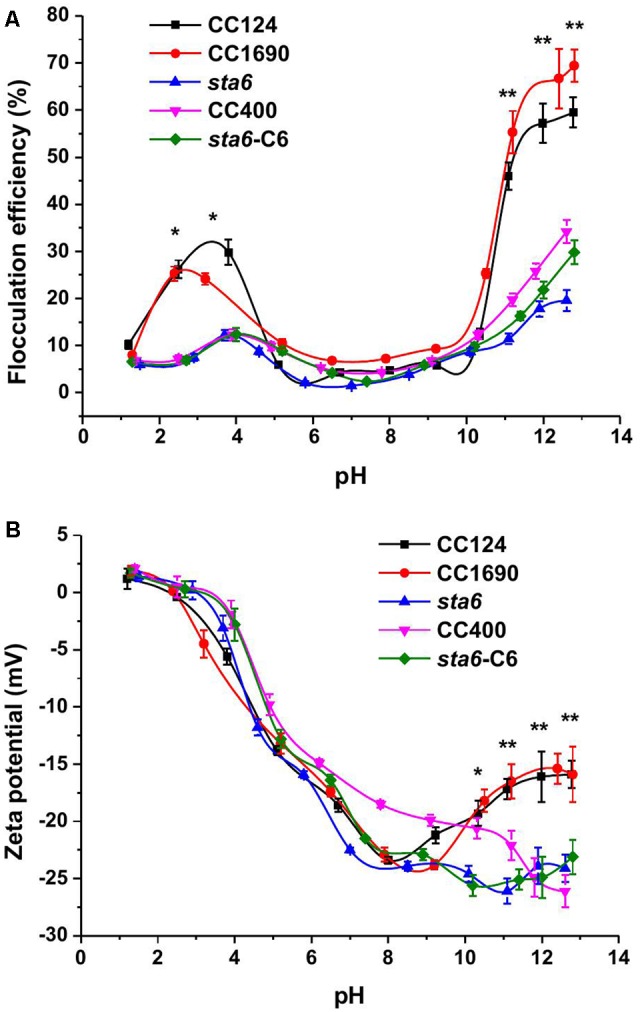
Flocculation efficiency and charge characteristics (Zeta potential) as a function of pH in three *Chlamydomonas reinhardtii* strains. **(A)** flocculation efficiency; **(B)** zeta potential. Sometimes the data points are not aligned, as it is quite difficult to adjust the same pH values in the solution for each strain. The data was analyzed for significant differences using the Student’s *t*-test (*n* = 3) (all mutants in comparison with the two wild-types). Asterisks indicate a significant difference from the control (^∗^*p* < 0.05, ^∗∗^*p* < 0.01).

The efficiency of cell flocculation in culture was first investigated as a function of pH (**Figure 1A**). Since an increase in metal precipitates would occur at the higher pH values and impact the OD measurements, the flocculation efficiency was calculated according to the absolute cell number. Less than 10% of flocculation effect on cell were observed when the pH of the cultures was varied between 5 and 9 for all measured strains. In this pH range, the flocculation efficiency was less than 10% for all five of the Chlamydomonas strains. However, the flocculating efficiency increased for CC124 and CC1690 in both the acidic and alkaline pH range. In the pH range of 11–13, the flocculation efficiency was highest for the cell wall-containing strains (45–70% for CC1690 and CC124) but relatively low (∼15–30%) with significant difference for the cell wall-deficient strains (CC400, *sta6* and *sta6*-C6). In the acidic range the flocculation efficiency was maximal at pH 4.0 for CC124 (32%) and pH 2.5 for CC1690 (26%). Overall, these results demonstrate that the highest flocculation efficiencies are obtained at high pH, although the efficiency values can be markedly different for the strains, probably a consequence differences in phenotypic characteristics, including the presence/absence of the cell wall (**Table 1** and **Figure 1A**).

Similar to the flocculation efficiencies, the zeta potential, which is widely used for quantification of the cell surface charge, was also pH dependent (**Figure 1B**). Over an 8.5-1.5 pH interval, the zeta potential showed a sharp increase from -24 to ∼0 mV. The profile of the zeta potential may have some link to flocculation efficiency; at pH 3–4, when the flocculation efficiency peaked, the cells were electrically near neutral. The zeta potential in the acidic region appeared unaffected by the presence/absence of a cell wall or by differences in cell size among the strains. However, at pH 10–13, the zeta potentials of both of wild-type strains moderately rose from ∼-25 to ∼-15 mV. In the three cell wall deficient mutants the zeta potential remained approximately the same (**Figure 1B**).

Several self-flocculation studies of microalgae involving pH adjustment of the growth medium have been performed (Wu et al., 2012; Liu et al., 2013). Increasing or decreasing the pH of the medium can increase the flocculation efficiency by up to 90% for green microalgae, suggesting an effective strategy for harvesting the cells. In our studies the cells were also subjected to a range of pH conditions, however, self-flocculation for wild-type cells was only relatively efficient at elevated pH (more than 10), and was never very high for cell wall deficient and starchless mutant cells (**Figure 1A**).

### Cation-Induced Flocculation

Tris-Acetate-Phosphate medium has high ionic strength with initial metal concentrations of Fe (∼1.0 mg/L), Ca (∼18.2 mg/L), and Mg (∼9.7 mg/L); after growth and pH adjustment, most of the metal salts were taken up and utilized by the algal cells. The background concentrations after flocculation were about 0.11, 1.83, and 0.94 mg/L for Fe, Ca, and Mg, respectively. Hence, it is likely that some of the flocculation elicited at high pH in the absence of additional metals (**Figure 1A**) is due to the presence of low concentrations of metal ions still present in the TAP media.

**FIGURE 2:**
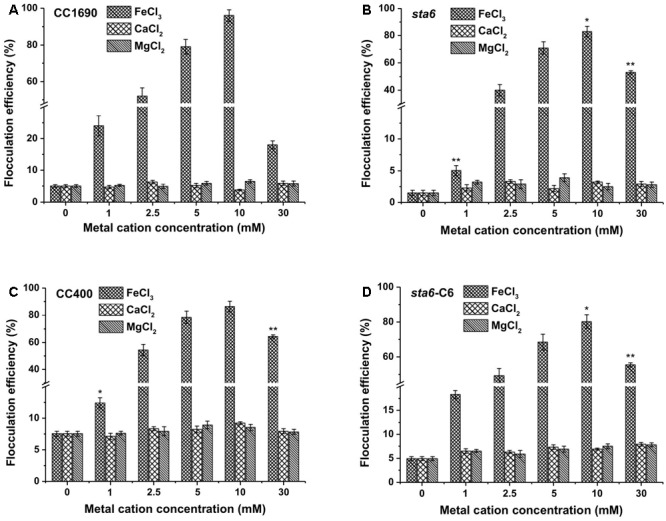
Flocculation efficiency as a function of different metal cations and their concentrations in wild-type and cell wall-deficient *Chlamydomonas reinhardtii* strain at near neutral pH conditions. **(A)** Wild-type CC1690; **(B)** cell wall deficient mutant *sta6*; **(C)** CC400; **(D)** *sta6-*C6. Cells were grown to stationary phase and the pH of the medium was ∼8.5. The data was analyzed for significant differences using the Student’s *t*-test (*n* = 3) (each mutant in comparison with wild-type). Asterisks indicate a significant difference from the control (wild-type) (^∗^*p* < 0.05, ^∗∗^*p* < 0.01).

We selected the divalent (Ca^2+^ and Mg^2+^) and trivalent (Fe^3+^) cation metals for further investigating the potential for harvesting Chlamydomonas cells by flocculation. We first used cells cultured in TAP medium and grown to stationary phase, with the pH of the medium for all of the cultures reaching ∼8.5. As shown in **Figure 2**, divalent cations (Ca^2+^, Mg^2+^) alone did not appear to impact flocculation efficiency beyond what was observed in the absence of supplementing cations (less than 10% after 10 min for the wild-type CC1690 (**Figure 2A**) and the various mutants (**Figures 2B–D**). The monovalent cation (K^+^) showed similar flocculation efficiencies as that of the divalent cations, although the resulting flocculants appeared more susceptible to disaggregation (data not shown). Although flocculation efficiencies following treatment with divalent cations were relatively low, the flocculation yields were somewhat higher in wild-type cells than in the mutant (5–6% for CC1690 compared to 2–3% for *sta6*; compare panel a to panel b in **Figure 2**); this could be explained by phenotypic differences such as cell size and/or motility (**Table 1**). In contrast, the trivalent ferric ion elicited strong flocculation, with efficiencies at 10 mM Fe^3+^ exceeding 95 and 85% for CC1690 and *sta6*, respectively. A further increase in the Fe^3+^ concentration to 30 mM caused a marked drop in flocculation efficiency to ∼18 and 50% for CC1690 and *sta6*, respectively (**Figures 2A,B**).

Since the pH adjustment and some cations were effective in eliciting self-flocculation for wild-type cells (**Figures 1A, 2**), we further explored flocculation using different metal cations at a range of pH conditions and quantified the levels of cations in the medium before and after flocculation at three different pHs (**Table 2**). The concentrations of Fe^3+^, Ca^2+^, and Mg^2+^ in the medium remained almost unchanged before and after cell flocculation at pH 4.0. However, following flocculation at pH 8.5, ∼25% of the Fe^3+^ and 72–84% of the Ca^2+^ and Mg^2+^ remained in the medium. Finally, cell flocculation at pH 12.0 resulted in precipitation of the majority of the metal ions from the medium, i.e., only 9.6–11.2%, 1.5–2.3%, and 18.2–29.3% of Fe^3+^, Ca^2+^, and Mg^2+^ remained in the medium, respectively. These results suggest that precipitation of the metal ions during flocculation occurred when the conditions were alkaline and possibly explain the relatively high flocculation efficiencies when such conditions are applied. Moreover, while precipitation of multivalent metal cations may co-occur with cells during flocculation at high pH, flocculation at low pH does not appear to be accompanied by metal ion precipitation and as a consequence, the low pH medium after flocculation still contains high cation levels and could not be used for the initiation of new cultures (Liu et al., 2013). Furthermore, metal cation precipitation during flocculation of three cell wall deficient mutants (*sta6*, CC400, and *sta6*-C6), all of which are also lacking flagella (**Table 1**), was generally no significant difference (Student’s *t*-test, *p* > 0.05) with what was observed for CC1690 and CC124, under any of the conditions examined (**Table 2**).

**Table 2:** Metal cation concentrations (5 mM of chlorides addition to the medium) before (C_1_) and after (C_2_) flocculation for wild-type and cell wall deficient *Chlamydomonas reinhardtii* strains at different pH level.

Strains	Elemental types	Fe^3+^			Ca^2+^			Mg^2+^		
	pH level	4.0	8.5	12.0	4.0	8.5	12.0	4.0	8.5	12.0
CC124	C_1_ (mg/L)	244.2 ± 3.4			203.4 ± 4.9			125.1 ± 4.4		
	C_2_ (mg/L)	245.3 ± 3.8	66.5 ± 5.4 (27.2%)	25.2 ± 1.8 (10.3%)	202.6 ± 3.4	153.2 ± 2.6 (75.3%)	3.5 ± 0.9 (1.7%)	124.1 ± 4.7	91.6 ± 4.3 (73.2%)	29.5 ± 2.1 (23.6%)
CC1690	C_1_ (mg/L)	249.5 ± 4.6			201.1 ± 5.4			121.7 ± 3.2		
	C_2_ (mg/L)	244.8 ± 2.7	58.4 ± 4.9 (23.4%)	23.9 ± 2.7 (9.6%)	203.1 ± 4.2	143.5 ± 3.9 (71.4%)	3.1 ± 0.4 (1.5%)	130.5 ± 2.1	88.2 ± 6.3 (72.5%)	22.1 ± 3.2 (18.2%)
*sta6*	C_1_ (mg/L)	245.8 ± 2.9			211.3 ± 3.6			133.8 ± 3.5		
	C_2_ (mg/L)	246.4 ± 3.7	65.3 ± 3.6 (26.6 %)	27.6 ± 4.4 (11.2%)	205.3 ± 4.7	177.8 ± 4.6 (84.1%)	4.9 ± 0.7 (2.3%)	127.4 ± 3.9	105.4 ± 5.8 (78.8%)	39.2 ± 4.9 (29.3%)
CC400	C_1_ (mg/L)	250.1 ± 3.3			209.3 ± 2.9			129.7 ± 3.9		
	C_2_ (mg/L)	252.2 ± 4.3	62.3 ± 3.9 (24.9%)	26.4 ± 4.2 (10.6%)	207.1 ± 5.2	172.3 ± 4.1 (82.3%)	4.8 ± 0.5 (2.3%)	130.5 ± 4.4	99.3 ± 5.4 (76.6%)	34.5 ± 3.9 (26.6%)
*sta6-*C6	C_1_ (mg/L)	248.4 ± 3.9			207.4 ± 3.2			131.4 ± 4.7		
	C_2_ (mg/L)	249.9 ± 4.2	65.9 ± 3.6 (26.5%)	29.3 ± 4.1 (11.8%)	204.3 ± 4.8	180.2 ± 2.7 (86.9%)	5.8 ± 0.6 (2.8%)	134.3 ± 4.1	106.3 ± 5.1 (80.9%)	37.4 ± 3.7 (28.5%)

*Numbers in brackets represent the percentage of remaining cation in the medium.*

### Using Coagulation-Flocculation Induced by High pH for Harvesting

**FIGURE 3:**
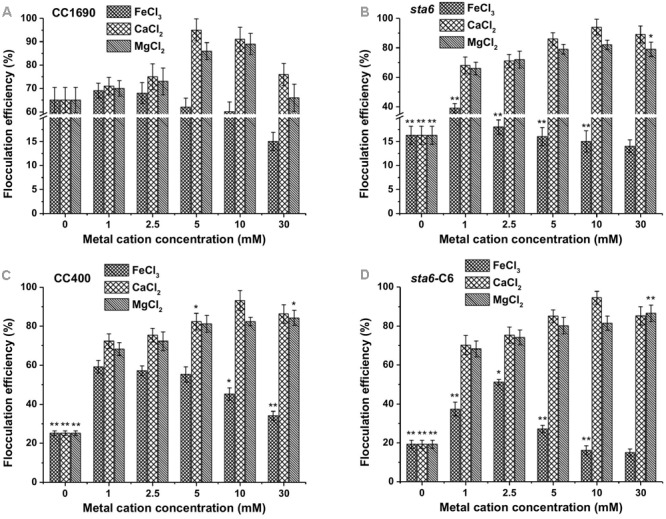
Flocculation efficiency as a function of different metal cations and their concentrations in wild-type and cell wall deficient *Chlamydomonas reinhardtii* strains at pH 11.5. **(A)** Wild-type CC1690; **(B)** cell wall deficient mutant *sta6*; **(C)** CC400; **(D)** *sta6-*C6. Cells were grown to stationary phase and the pH of the medium was ∼11.5. The data was analyzed for significant differences using the Student’s *t*-test (*n* = 3) (each mutant in comparison with wild-type). Asterisks indicate a significant difference from the control (wild-type) (^∗^*p* < 0.05, ^∗∗^*p* < 0.01).

Since the flocculation efficiencies and charge characteristics as a function of pH were similar between CC1690 and CC124 (both are *CW*^+^), we analyzed CC1690 as a ‘wild-type’ representative for additional experiments. Since the highest flocculation efficiencies were observed at a high pH (**Figure 1A**), we evaluated the impact of metal cation concentrations at pH 11.5 on the flocculation efficiencies of both CC1690 and *sta6*. The flocculation efficiencies of CC1690 and *sta6* peaked at 5–10 mM Ca^2+^ and Mg^2+^ (**Figures 3A,B**), with the highest flocculating activities resulting in aggregation of 90–95% of the cells (**Table 1**). For all divalent cations used in our study, the flocculation efficiencies either declined to some extent or remained the same when the ion concentration was elevated to 30 mM. Furthermore, Mg^2+^ was slightly less effective at eliciting aggregation than Ca^2+^ (**Figure 3** and **Supplementary Figure S2**), and the highest flocculation efficiencies, when using Mg^2+^, were 89.1 and 81.9% for CC1690 and *sta6*, respectively. In contrast, at pH 11.5 the ferric ions generally caused some increase in flocculation at 1–5 mM, with a negative impact at higher concentrations (10 and 30 mM); at 1 mM Fe^3+^, the maximal flocculation efficiencies were 69.2 and 39.3%, for CC1690 and *sta6*, respectively. Additionally, as a consequence of flocculation with Fe^3+^, both the cells and supernatant turned sandy beige, while for the cultures that were subjected to Ca^2+^- and Mg^2+^-stimulated flocculation, a change in medium color did not occur (**Supplementary Figure S3**).

Previous studies have demonstrated that flocculation elicited by lowering the pH is likely the result of neutralizing of the negative surface charges of the cells by protonation of carboxyl and/or sulfate groups (Wyatt et al., 2012; Liu et al., 2013). However, in this work we demonstrated that cell flocculation occurs at high pH using Ca^2+^ and Mg^2+^ ions, and therefore the flocculation reaction is likely to result from a chemical precipitation of the calcium and/or magnesium salts (Vandamme et al., 2012; Beuckels et al., 2013). Indeed, self-flocculation greatly reduced the level of metal cations in the medium (**Table 2**). Hence, our results confirm that coagulation-flocculation could be induced by the addition of metal salts (**Figure 3** and **Supplementary Figure S2**), and that high pH (greater than 10) is required for this process to occur (**Table 1**).

A recent study showed that 83% of the cell wall deficient Chlamydomonas *cw15* cells that were nitrogen deprived flocculated when supplemented with Ca^2+^ under slightly alkaline conditions (Scholz et al., 2011). Moreover, Scholz et al. (2011) demonstrated that fresh TAP medium was less active in eliciting cell flocculation than nitrogen-free TAP medium. These results suggest that a flocculation inhibitory component of the medium was consumed during growth and/or that a metabolic byproduct released into the medium by nitrogen starved cells promotes flocculation (Scholz et al., 2011).

### The Impact of Starch Accumulation on Flocculation

Since we compared the ‘wild-type’ *cw*^+^ (CC1690) with the *sta6 cw*^-^ starchless mutant, we sought to clarify whether or not cellular starch impacts flocculation efficiency. To address this issue we compared *sta6* to two *cw*^-^ strains, CC400 (widely used wall-deficient mutant) and CC4567 (*sta6*-C6) (complemented *sta6* created by transformation of BAFJ5 with the plasmid pSL-STA6 harboring a genomic copy of the wild-type *STA6* gene) that are not impaired in starch production (Li et al., 2010). Importantly, the average size of *sta6* was smaller than CC400 and *sta6*-C6 (**Supplementary Figure S2**) and the cellular components, such as total lipids and starch contents among the strains varied from 11–45% to 0–39%, respectively (**Table 1**), with the highest lipid and no detectible starch in *sta6*. Despite differences in cells size and content of organic polymers, no substantial difference of Zeta potential was observed among the different strains (**Table 1**). Cation content in the medium derived from flocculated cells from each strain at a given pH exhibited similar levels (**Table 2**), although the level of cation in the medium was highly dependent on the pH value.

Flocculation efficiency at different divalent cation concentrations (pH 11.5) showed no significant difference between CC1690, CC400, *sta6-*C6, and *sta6*; all peaked at 5–10 mM Ca^2+^ and Mg^2+^ (with highest flocculating efficiencies of around 94 and 80%, respectively) (**Figures 3C,D**). These results suggest that cellular starch content has little overall impact on Chlamydomonas flocculation.

In this study, differences in flocculation efficiencies observed for wild-type and mutant cells are probably explained by the absence/presence of a cell wall and/or flagella. Intuitively, cells such as *sta6*, which lack flagella, might be thought to be more amenable to aggregation than motile cells since they might be more susceptible to the metal salt coagulants formed under strong alkali conditions. In addition, the loss of starch synthesis in *sta6* does not cause re-direction of fixed carbon to the synthesis of more protein or lipid; the *sta6* culture appears to accumulate ∼20% less biomass (because the cells are smaller) when grown in nutrient-replete medium (Krishnan et al., 2015), even though the cell density is higher. However, it was also shown that following growth under some stress conditions, *sta6* cells could accumulate more lipids, which makes them float to the surface of the culture (Goodenough et al., 2014). Based on the flocculation features of *sta6* reported here, we suggest that this strain can be effectively used for future biofuel research and production.

### Reuse of Growth Medium

**FIGURE 4:**
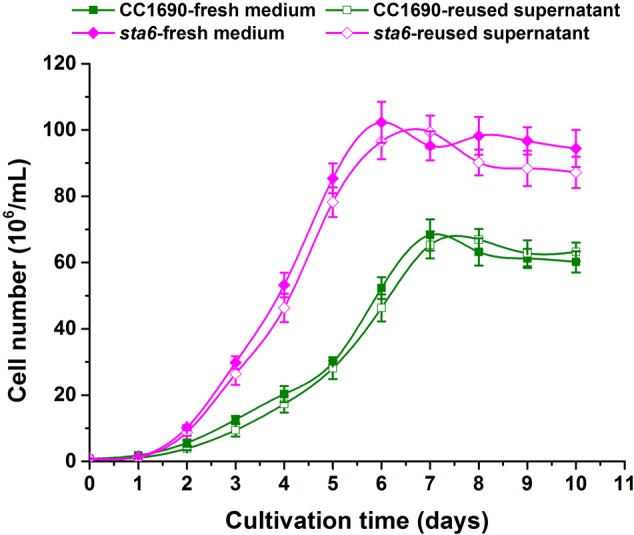
Comparison of cell growth as a function of fresh and recycled medium.

A highly important consideration in a large scale industrial application is the capacity to reuse the spent growth medium. The metal cation concentrations that remain in the medium following cell flocculation can have a marked and direct impact on media re-cycling capabilities. We therefore assessed the recyclability of the spent medium following photoautotrophic cultivation. Using 5–10 mM calcium with cultures of CC1690 and *sta6* we achieved efficient flocculation (>90%; **Figure 3**) and then determined whether the used medium affected the growth of new cultures. To address this, we compared the CC1690 and *sta6* growth rates over a period of 10 days in fresh and recycled medium. At any given time point the growth rate (for both CC1690 and *sta6*) was similar regardless of whether the cells were cultured in fresh or recycled medium (**Figure 4**). Moreover, both strains maintained their ability to flocculate with the addition of Ca^2+^ to the medium as long as there was a near neutral or alkaline environment during cultivation. Thus, residual Ca^2+^ or coagulant does not appear to be an obstacle to medium reuse. We also observed that cells grown in the spent supernatant, which contained a small quantity of residual Ca^2+^, required less Ca^2+^ to flocculate. However, the amount of Ca^2+^ salts that precipitate was likely higher in the flocculated biomass; further analysis is required to evaluate whether the Ca^2+^ that precipitates with the flocculated cells interferes with specific microalgal biomass applications, e.g., biofuels production or bio-refinery.

## Conclusion

Our experiments demonstrated that self-flocculation by direct pH adjustment does not work well for Chlamydomonas and that it depends on the presence of sufficiently high metal salts (>5 mM). The highest flocculation efficiency was achieved using a combination of added Ca^2+^ with elevated pH (>10). Slightly more metal cation appears to be needed to flocculate *sta6* (cell wall deficient, starchless mutant) potentially owing to the lack of motility, the different surface structure or the smaller cell size (Sathe and Durand, 2016). We also confirmed that cellular starch content has little overall impact on flocculation of Chlamydomonas, and importantly, the flocculated medium could be re-reused. Further examination of the flocculation mechanism would entail examining additional differences in flocculation characteristics among cells with different phenotypes, the impact of metal salts on flocculation when the culture is scaled up and whether or not the findings apply to other algal groups.

Additional research on the conditions (e.g., pH and metal ions) used for efficient flocculation of diverse algal groups with diverse characteristics, at both small and large scale, would help establish inexpensive procedures for harvesting cell biomass.

## Author Contributions

Conceived and designed the experiments: JF. Performed the experiments: JF and LZ. Analyzed the data: JF, SS, YB, and AG. Wrote the paper: JF, SS, YB, and AG.

## Conflict of Interest Statement

The authors declare that the research was conducted in the absence of any commercial or financial relationships that could be construed as a potential conflict of interest.

## Footnotes

1 https://www.chlamycollection.org/methods/media-recipes/tap-and-tris-minimal/

## Supplementary Material

FIGURE S1 Cell growth of different Chlamydomonas strains.

FIGURE S2 Light Microscopy of Chlamydomonas cells. **(a)** CC1690; **(b)** CC124; **(c)** *sta6*; **(d)** CC400; **(e)** *sta6*-C6. Scale bars: 10 μm.

FIGURE S3 Microscopic images of Chlamydomonas cells flocculated by metal cations and elevated pH (11.5). **(a)** Wild-type CC1690 before treatment; **(b)** wild-type CC1690 after treated with 5 mM FeCl_3_; **(c)** wild-type CC1690 after treated with 5 mM CaCl_2_; **(d)** wild-type CC1690 after treated with 5 mM MgCl_2_; **(e)** cell wall deficient *sta6* mutant before treatment; **(f)** *sta6* mutant after treatment with 5 mM FeCl_3_; **(g)** *sta6* mutant after treated with 5 mM CaCl_2_; **(h)** *sta6* mutant after treated with 5 mM MgCl_2_. Scale bars: 10 μm.
