# Supplementary figures and images for: Flocculation of Chlamydomonas reinhardtii with Different Phenotypic Traits by Metal Cations and High pH
Source: Front Plant Sci. 2017 Nov 20;8:1997. doi: 10.3389/fpls.2017.01997 (PMC5702007; doi:10.3389/fpls.2017.01997)

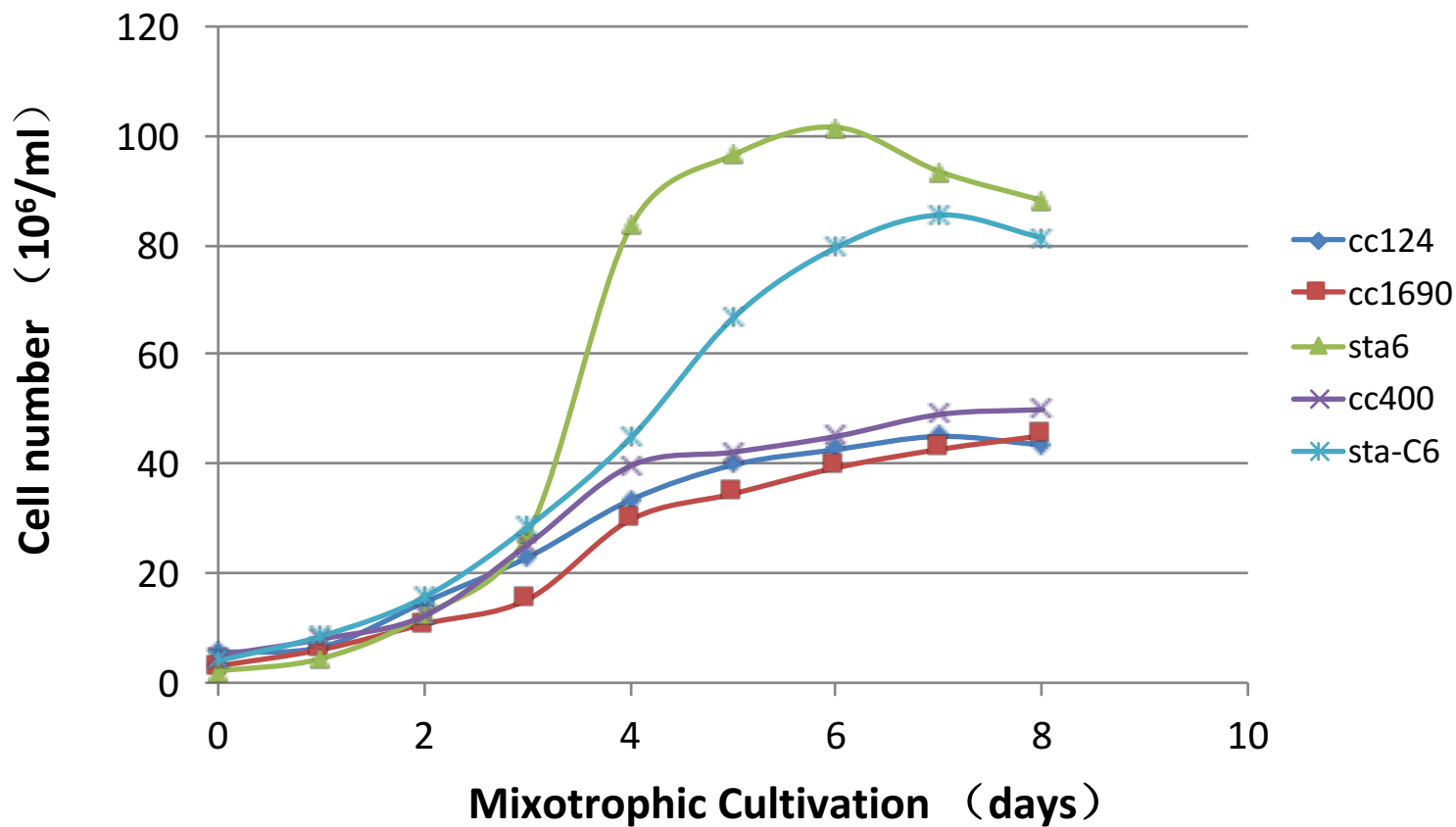

Supplement: FIGURE S1 — Cell growth of different Chlamydomonas strains. [file Image_1.PDF]

**a**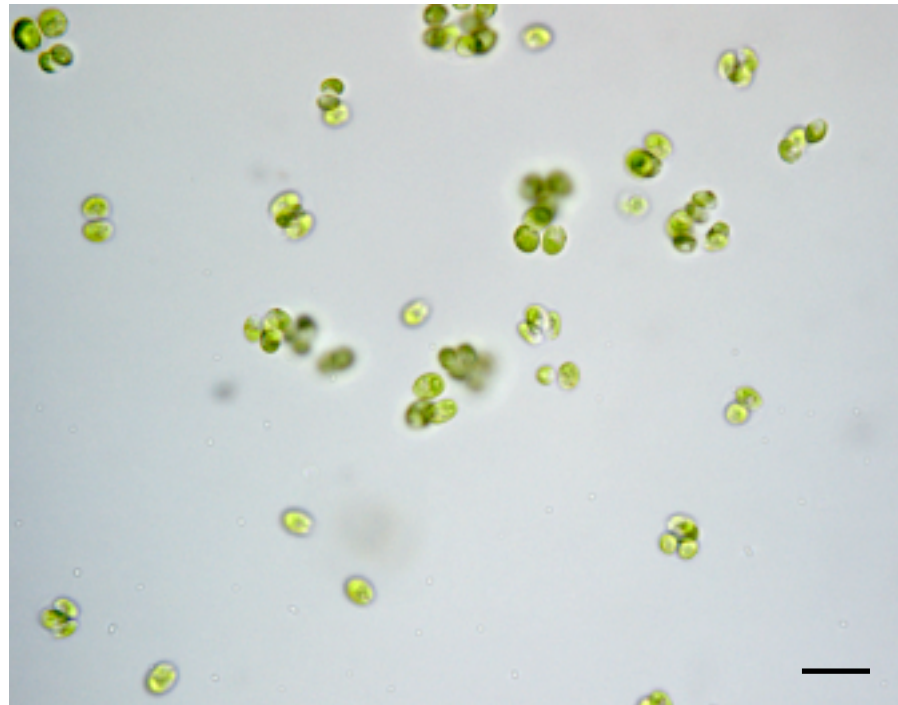

CC1690 ( $6.89 \pm 0.25 \mu\text{m}$ )

**b**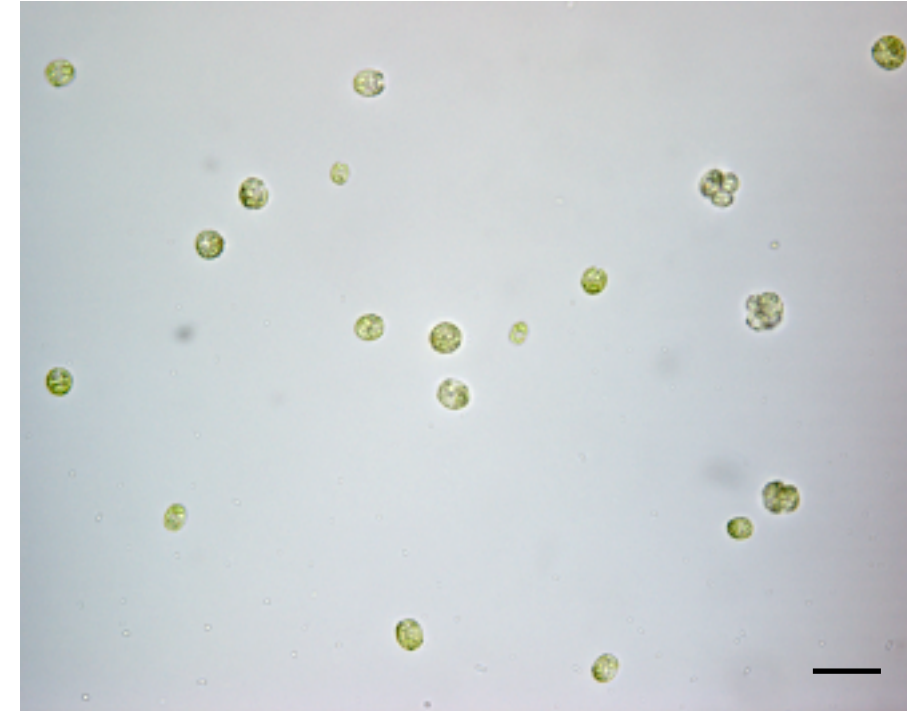

CC124 ( $5.96 \pm 0.37 \mu\text{m}$ )

**c**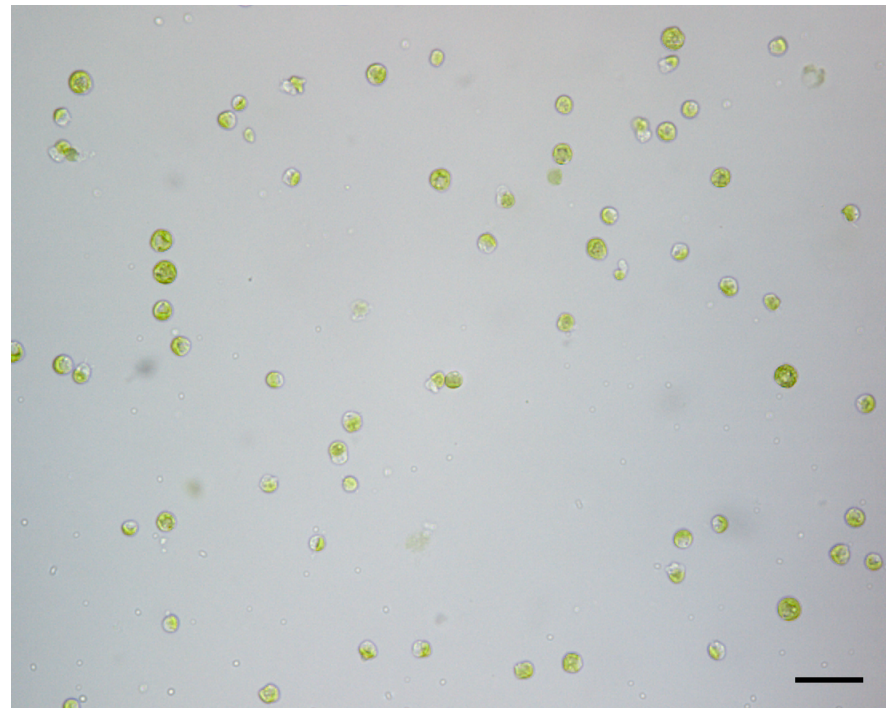

*Sta6* ( $2.33 \pm 0.18 \mu\text{m}$ )

**d**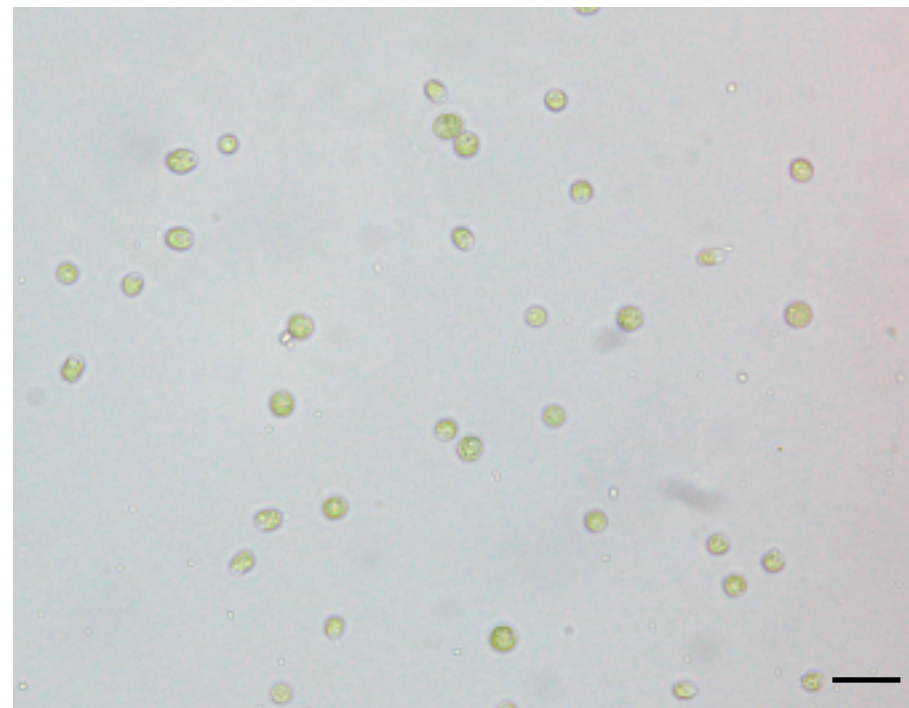

CC400 ( $4.27 \pm 0.24 \mu\text{m}$ )

**e**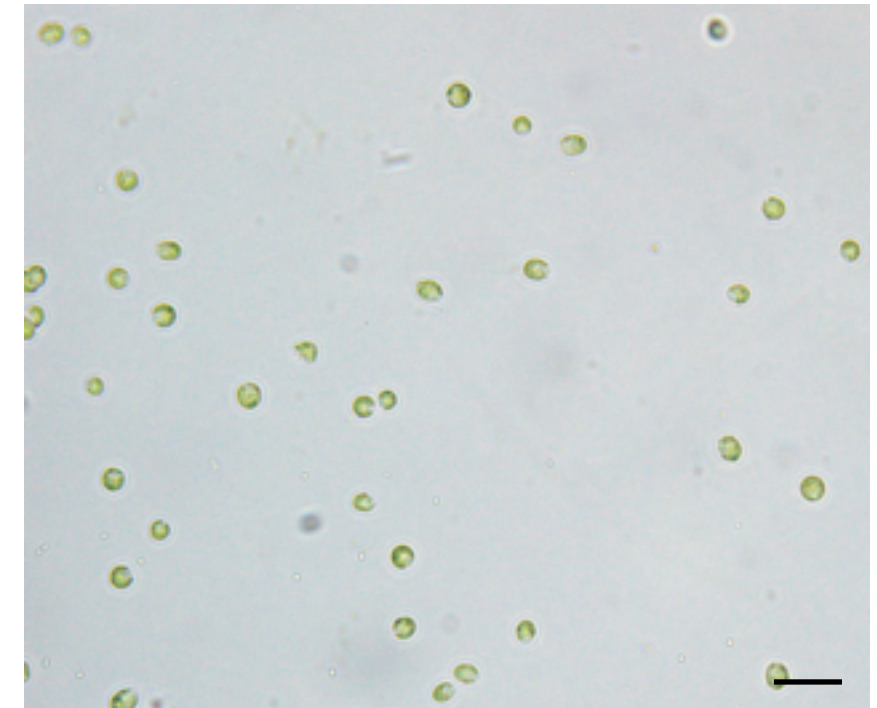

*Sta6-C6* ( $3.57 \pm 0.21 \mu\text{m}$ )

Supplement: FIGURE S2 — Light Microscopy of Chlamydomonas cells. (a) CC1690; (b) CC124; (c) sta6; (d) CC400; (e) sta6-C6. Scale bars: 10 μm. [file Image_2.PDF]
