# Supplementary material for: Flocculation of Chlamydomonas reinhardtii with Different Phenotypic Traits by Metal Cations and High pH
Source: Front Plant Sci. 2017 Nov 20;8:1997. doi: 10.3389/fpls.2017.01997 (PMC5702007; doi:10.3389/fpls.2017.01997)
Supplement: FIGURE S3 — Microscopic images of Chlamydomonas cells flocculated by metal cations and elevated pH (11.5). (a) Wild-type CC1690 before treatment; (b) wild-type CC1690 after treated with 5 mM FeCl3; (c) wild-type CC1690 after treated with 5 mM CaCl2; (d) wild-type CC1690 after treated with 5 mM MgCl2; (e) cell wall deficient sta6 mutant before treatment; (f) sta6 mutant after treatment with 5 mM FeCl3; (g) sta6 mutant after treated with 5 mM CaCl2; (h) sta6 mutant after treated with 5 mM MgCl2. Scale bars: 10 μm. [file Image_3.PDF]

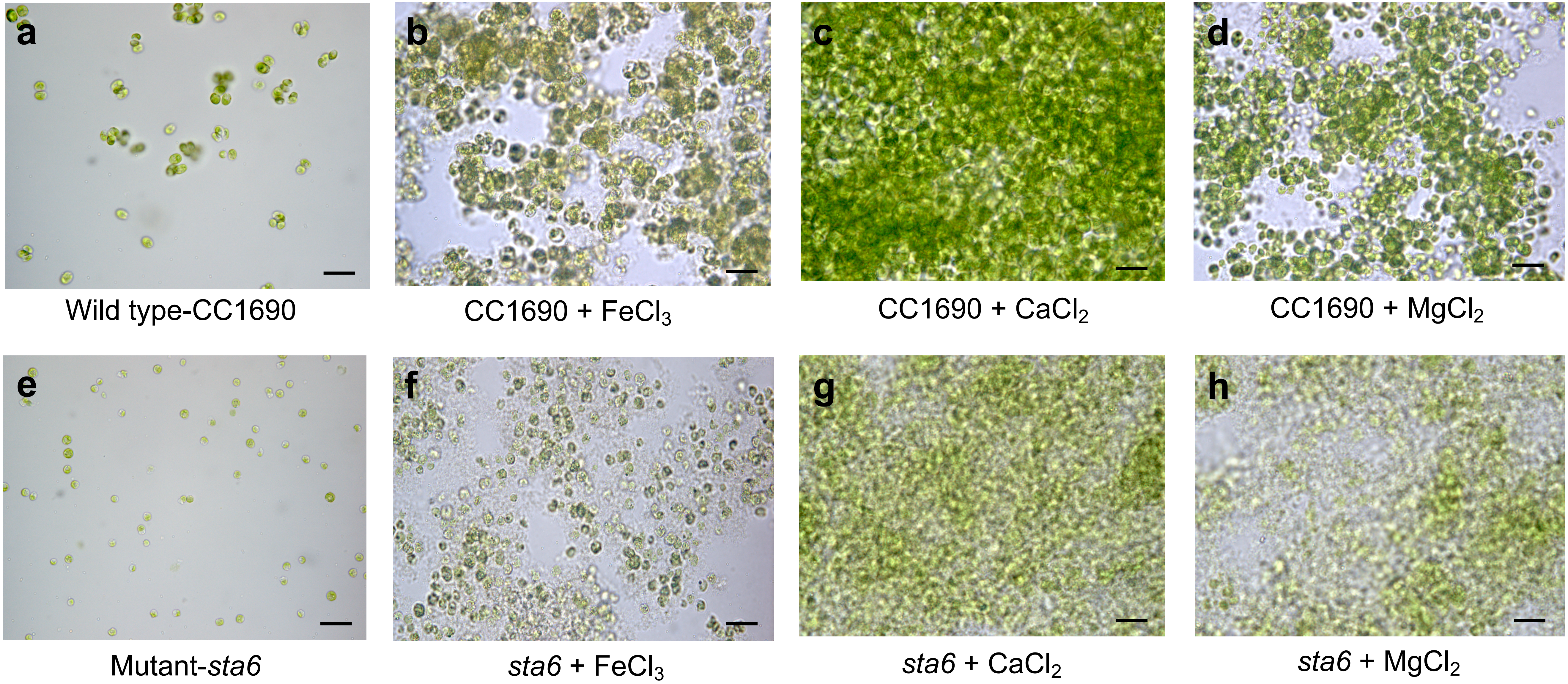
a
b
c
d
Wild type-CC1690
CC1690 + $FeCl_3$
CC1690 + $CaCl_2$
CC1690 + $MgCl_2$
e
f
g
h
Mutant-*sta6*
sta6 + $FeCl_3$
sta6 + $CaCl_2$
sta6 + $MgCl_2$
