# Supplementary material for: Flocculation of Chlamydomonas reinhardtii with Different Phenotypic Traits by Metal Cations and High pH
Source: Front Plant Sci. 2017 Nov 20;8:1997. doi: 10.3389/fpls.2017.01997 (PMC5702007; doi:10.3389/fpls.2017.01997)
Supplement: Supplementary file 4 [file Table_1.DOCX]

**Table S1. Strains used in this study.**

| **Strains** | **Note** | **Background** |
| --- | --- | --- |
| CC1690 | intact cell wall;  flagella-dependent motility;  able to produce starch | *mt^+^ NIT1* *NIT2* |
| CC124 | intact cell wall;  flagella-dependent motility;  able to produce starch | 137c *mt^-^ nit1 nit2* |
| *sta6* | cell wall deficient;  lacking flagella-dependent motility;  unable to accumulate starch | *cw15 arg7-7 nit1 nit2 sta6-1::ARG7* |
| CC400 | cell wall deficient;  lacking flagella-dependent motility;  able to produce starch | *cw15* |
| *sta6-*C6 | cell wall deficient;  lacking flagella-dependent motility;  able to produce starch | *cw15 arg7-7 nit1 nit2 sta6-1::ARG7 STA6* |
